# Evaluation of the Mechanical and Physical Properties of Maxillofacial Silicone Type A-2186 Impregnated with a Hybrid Chitosan–TiO_2_ Nanocomposite Subjected to Different Accelerated Aging Conditions

**DOI:** 10.3390/biomimetics8070539

**Published:** 2023-11-11

**Authors:** Faten K. Al-Kadi, Jwan Fateh Adbulkareem, Bruska A. Azhdar

**Affiliations:** 1 Department of Prosthodontics, College of Dentistry, University of Sulaimani, Sulaymaniyah 46001, Iraq; jwan.abdulkareem@univsul.edu.iq; 2 Nanotechnology Research Laboratory, Department of Physics, College of Science, University of Sulaimani, Sulaymaniyah 46001, Iraq; bruska.azhdar@univsul.edu.iq

**Keywords:** synthesized chitosan–TiO_2_ nanocomposite, RTV of maxillofacial silicone, mechanical and physical properties, accelerated aging conditions

## Abstract

The effects of incorporating a pioneer chitosan–TiO_2_ nanocomposite on the mechanical and physical properties of room-temperature vulcanization (RTV) maxillofacial A-2186 silicone under accelerated aging protocols were rigorously examined. This investigation utilized 450 samples distributed across five distinct silicone classifications and assessed their attributes, such as tensile strength, elongation, tear strength, hardness, and surface roughness, before and after various accelerated aging processes. Statistical methodologies, including a one-way ANOVA, Tukey’s HSD, and Dunnett’s T3, were employed based on the homogeneity of variance, and several key results were obtained. Silicones infused with 1 wt.% chitosan–TiO_2_ showed enhanced tensile strength across various aging procedures. Moreover, the 1 wt.% TiO_2_/Chitosan noncombination (TC) and 2 wt.% TiO_2_ compositions exhibited pronounced improvements in the elongation percentage. A consistent rise was evident across all silicone categories regarding tear strength, with the 1 wt.% chitosan–TiO_2_ variant being prominent under certain conditions. Variations in hardness were observed, with the 1 wt.% TC and 3 wt.% chitosan samples showing distinctive responses to certain conditions. Although most samples displayed a decreased surface roughness upon aging, the 1 wt.% chitosan–TiO_2_ variant frequently countered this trend. This investigation provides insights into the potential of the chitosan–TiO_2_ nanocomposite to influence silicone properties under aging conditions.

## 1. Introduction

The importance of patient satisfaction and the assessment of quality of life (QOL) is increasing in the realm of healthcare quality. The success of treatments and the degree of patient reintegration are predominantly determined through subjective patient evaluations. Nevertheless, research findings consistently demonstrate significant enhancements in QOL following maxillofacial prosthetic treatment. This highlights the substantial positive influence of these interventions on patients’ overall wellbeing and the successful reintegration into their daily lives [1].

In addition to protecting areas with exposed and bleeding tissues resulting from surgical resections, traumas, tumors, or congenital issues, maxillofacial prostheses offer a non-invasive and risk-free treatment option for esthetic restoration. These prostheses play a crucial role in enhancing self-esteem, improving quality of life, and facilitating a successful reintegration of a patient into society [2,3].

The ideal properties sought in a material intended for use as a maxillofacial prosthetic material include superior tear strength, tensile strength, and elongation at break. Also, favorable surface wettability, low hardness, and water absorption are desired. These attributes collectively contribute to the material’s suitability for practical and comfortable use in maxillofacial prosthetic applications [4].

Maxillofacial prosthetic materials exhibit a comprehensive array of chemical structures, thereby resulting in a diverse spectrum of physical properties. These properties vary significantly, ranging from the hardness and stiffness of alloys and polymers to the flexibility of elastomers and soft polymers. Examples of these materials include latexes, poly (methyl methacrylate) (PMMA), poly(vinyl chloride) (PVC), polyurethanes, and silicone rubber materials [5].

Maxillofacial prostheses are predominantly constructed using silicone as the primary material due to its advantageous characteristics [6,7,8,9]. Silicone offers flexibility, promoting the patient’s overall wellbeing and comfort. Moreover, it possesses properties such as a texture that closely resembles human skin; stability, when subjected to heat; and the ability to prevent bacterial colonization by repelling water, blood, and organic substances. These features make silicone an ideal choice for fabricating maxillofacial prostheses, ensuring optimal functionality and patient satisfaction [1,10,11].

Despite its advantages, silicone has limitations, particularly regarding early material deterioration. Silicone prostheses may experience issues within one to three months, such as modified texture, ill-fitting edges due to shape changes, decreased tear strength, and material discoloration. These factors highlight the need for regular maintenance and replacement to ensure the longevity and effectiveness of silicone-based maxillofacial prostheses [11,12,13,14,15].

Silicone’s susceptibility to discoloration is a significant drawback that significantly impacts the lifespan of prostheses. Discoloration can occur due to exposure to external factors, including sunlight, artificial UV light, air pollution, cosmetics, temperature fluctuations, natural climate conditions, and human body secretions [16,17,18]. Additionally, the highly permeable nature of silicone can lead to discoloration when various disinfection procedures are utilized as part of the prosthesis maintenance routine [19].

The maxillofacial prosthesis can absorb sweat and sebum as it remains in contact with the human skin over time. However, the exposure to UV radiation poses significant challenges. While UV radiation enhances crosslinking within silicone, it also leads to the breakdown of bonds in the polymer matrix, causing a decelerated polymerization rate and degradation of the silicone material. As a consequence of these processes, the prosthesis undergoes color changes and experiences material deterioration at an accelerated rate [20].

Numerous studies have demonstrated the efficacy of various nanoparticles in enhancing the color stability of the silicone elastomers used in facial prostheses. These nanoparticles have been found to effectively block UV rays, resulting in improved color stability. Additionally, their use has shown positive effects on the hardness, tear strength, tensile strength, percentage elongation, and antifungal properties of silicone elastomers [21].

Nanoparticles function as UV protectants because their dimensions are smaller than the UV light wavelength. Upon exposure to this radiation, the electrons within these particles oscillate, leading them to scatter a light segment and absorb another. As a result, the finer the nanoparticles, the more effective their defense against solar radiation. In a more comprehensive context, particles at the nano-scale manifest unique physical, chemical, and biological characteristics relative to their macro-scale equivalents, predominantly attributed to their pronounced surface area-to-volume ratio. The properties of nanoparticles are contingent upon their size and concentration [22].

Adding nanosized TiO_2_ and ZnO (inorganic white powders) to polymer materials can significantly improve their mechanical and optical properties. This is primarily due to the unique characteristics of nanoparticles, such as their small size, large specific surface area, active functional groups, and strong interfacial interaction with organic polymers. By incorporating these nanoparticles, the physical and optical properties of polymers can be enhanced while also providing resistance against aging caused by environmental stresses. In the case of silicone maxillofacial elastomers, nanosized TiO_2_ has been found in the research to be the most effective opacifier, with concentrations of 2.0 wt.% and 2.5 wt.% by weight yielding the best opacity and color stability results [23]. According to a study conducted by Akay et al. [21], the addition of nanoparticles, such as TiO_2_, fumed silica, and silane silica, to a commercially available silicone-based elastomer used for maxillofacial prostheses fabrication was observed to be non-toxic. TiO_2_ is a photocatalyst with a high UV absorbance capacity across a broad range of wavelengths, making it a suitable active ingredient in sunscreen cosmetics [24].

In the research, incorporating nano-TiO_2_ into both room-temperature-vulcanizing (RTV) and high-temperature-vulcanizing (HTV) maxillofacial silicone elastomers has been shown to enhance specific mechanical properties of silicone materials, including the tear strength, tensile strength, and elongation at break. Furthermore, the addition of nano-TiO_2_ is directly proportional to an increase in the hardness of the silicone material [25]. However, it is crucial to note that the concentration of nano-TiO_2_ should not exceed 2.0 wt.%. Beyond this concentration, the agglomeration of nanoparticles may occur, reducing the material’s mechanical strength and UV shielding efficiency [23].

An alternative approach to address these limitations involves using composite materials comprising a support material called TiO_2_. Recently, considerable attention has been directed towards investigating the synergistic effects of incorporating TiO_2_ nanoparticles into chitosan coatings, aimed at enhancing the properties of nanocomposite films. These properties encompass mechanical strength, swelling characteristics, and thermal stability. Chitosan ensures the effective dispersion of metal oxide particles, preventing aggregation and promoting compatibility between components [26].

The pharmaceutical industry has recently shown a keen interest in chitosan due to its remarkable properties, such as biodegradability, biocompatibility, non-toxicity, non-immunogenicity, and strong bioadhesive characteristics. These features have made chitosan a highly sought-after compound as an antibacterial and antifungal agent in pharmaceutical applications [27]. Chitosan is derived from chitin through N-deacetylation and is widely acknowledged in the field as a versatile and biocompatible biomaterial. Among the natural polymers, chitosan is the most extensively utilized. Numerous in vivo studies have demonstrated chitosan derivatives’ non-toxic nature and excellent biocompatibility characteristics, particularly CM-chitosan [28]. Chitosan functions as a modifier or enhancer for TiO_2_, which is analogous to a dopant, even though conventional metal dopants typically attract electrons generated during the process of photocatalysis. Chitosan’s role deviates from the conventional electron attraction mechanism; however, in the research, its presence has been observed to positively influence the photocatalytic performance of TiO_2_ [24].

Combining polymers is a straightforward approach to creating innovative materials that maintain the essential qualities of the base polymer. It is common for these polymer mixtures to be incompatible, resulting in a granular structure due to the stress between the combined phases. A study conducted by Remanan et al. showed that, through the ultrasonic integration of a TiO_2_/chitosan/GO nanocomposite filler, the resulting membrane notably improved its defense against bacterial adherence and fouling [29].

Despite the prevalence of numerous studies investigating the consequences of adding nanoparticles to maxillofacial silicone and exposing it to different accelerated aging conditions [10,25,30,31,32,33], there is a significant gap in the research specifically focusing on the effects of integrating the hybrid chitosan–TiO_2_ nanocomposite and its influence on the mechanical and physical properties of the maxillofacial silicone material.

The objective of this study was to examine how the inclusion of the chitosan–TiO_2_ nanocomposite affects the mechanical and physical properties of room-temperature-vulcanized (RTV) maxillofacial A-2186 silicone when exposed to diverse accelerated aging conditions.

The null hypothesis states that the mechanical and physical properties of the maxillofacial A-2186 silicone elastomer are not influenced by the integration of the chitosan–TiO_2_ nanocomposite, even after undergoing various accelerated aging procedures.

## 2. Materials and Methods

### 2.1. Materials

Four hundred and fifty samples were created using a room-temperature-vulcanized (RTV) maxillofacial silicone elastomer A-2186 (Factor II Inc., Lakeside, AZ, USA). The samples’ sizes were drafted using Auto CAD 2013 and crafted with a computer numerical control (CNC) machine. Molds for the specimens were manufactured from transparent acrylic sheets, each containing a base, frame, and cover, with all being precisely measured by the researcher. The molds were formed according to the required dimensions.

**Figure 1:**
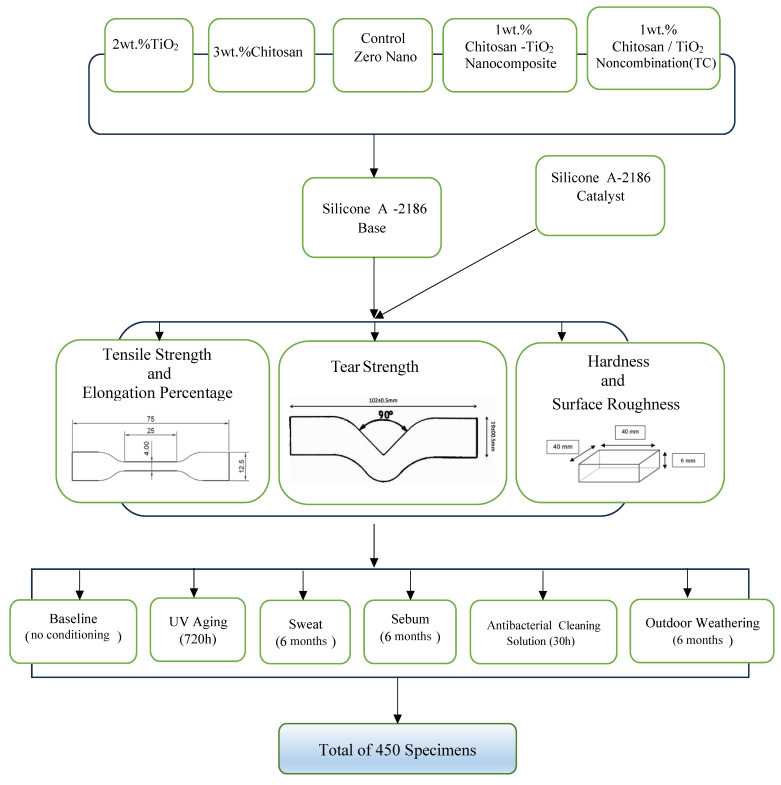
The flowchart of this study.

Five distinct classifications of silicone were methodically fabricated for this study. Figure 1 presents the study design. The first classification, the control, was composed of pure silicone without nanoparticle infusion. The second classification featured titanium dioxide nanoparticles incorporated into the silicone at a concentration constituting 2 wt.% [30,34] of the total weight. This was sourced from Sigma-Aldrich(Chemie GmbH Eschenstrasse 5 D-82024 TAUFKIRCHEN, Germany), identified by CAS Number 718467, and the nanoparticles possessed a primary particle size of 21 nm. The third classification incorporated chitosan, infused at a concentration of 3 wt.% by weight [22]. This substance was also obtained from Sigma-Aldrich under CAS Number 448869. The chitosan exhibited a degree of acetylation between 75% and 85% and a low molecular weight. The fourth classification was characterized by a hybrid nano-mixture of titanium dioxide (TiO_2_) and chitosan (TC), combined at a total concentration of 1% by weight, split equally with 0.5 wt.% of TiO_2_ and 0.5 wt.% of chitosan. The fifth and final classification involved the impregnation of silicone with a synthetically derived nanocomposite of chitosan–TiO_2_ powder at a 1% concentration by weight [35].

### 2.2. Methods

#### 2.2.1. Preparation of the Nanocomposite

In a characteristic application of the core–shell method [36], a quantity of two grams of titanium dioxide (TiO_2_) was dispersed in a solution containing 200 mL of 1% (*v*/*v*) acetic acid (CH_3_COOH) (obtained from Merck, Rahway, NJ, USA (Cat. No. 100063). The solution was subjected to sonication for thirty minutes at an ambient room temperature, utilizing a Q700 Sonicator from Qsonica LLC. This process facilitated the transformation of TiO_2_ into Ti^4+^ ions. Concurrently, 2 g of chitosan was dispersed into a separate solution containing 200 mL of 1% (*v*/*v*) acetic acid and then sonicated for thirty minutes at room temperature. Both solutions (TiO_2_ and chitosan, 200 mL each) were then combined and subjected to continuous stirring until a clear sol was achieved at room temperature. Subsequently, a solution of sodium hydroxide (NaOH) with a concentration of (1 M) was gradually introduced dropwise to the combined solution until the pH level reached ten. The solution was then separated, and the residual substance was filtered using a Buchner funnel. It was thoroughly rinsed with abundant distilled water until the pH level of the resultant chitosan–TiO_2_ solution stabilized at a pH of seven. Following the filtration and washing steps, the mixture was placed in a vacuum oven to dry at a temperature of 60°C overnight. To ensure complete dryness was achieved, the composite was further subjected to a drying process using a magnetic stirrer (LabTech LMS-1003; Daihan Labtech Co., Ltd., Namyangju-si, Republic of Korea) for two hours at 80 °C. Finally, the dried composite was pulverized using a mortar to produce a fine powder. The assessment of the morphological features was executed using the FESEM system, XRD, and FTIR.

#### 2.2.2. Preparation of Control Group Specimens

As per the manufacturer’s instructions, the RTV silicone type A-2186 was prepared in a mixing ratio of the base to the catalyst, set at 10:1. The mixing procedure for the control group commenced by adding the base into a container placed on an electronic balance, notable for its precision to 0.0001 grams (utilizing the Nimbus Analytical from Adam Equipment, Nimbus Analytical, Adam Equipment, Oxford, CT, USA). Subsequently, the catalyst was incorporated and thoroughly mixed for five minutes utilizing a vacuum mixer (Model AX-2000, produced by Aixin Medical Equipment Co., AX-2000, Aixin Medical Equipment Co., Ltd., Tianjin, China). This mixing process was conducted at a speed of 360 revolutions per minute and under a vacuum pressure of −0.09 bar.

The mixture was then placed in a vacuum chamber for fifteen minutes to eliminate any entrapped air bubbles that were present. Subsequently, the mixed material was left to stand undisturbed for three minutes in the chamber without a vacuum to enable the material to settle. The mixture was then poured into plastic molds. G-clamps were employed to ensure a secure fit, allowing any excess silicone material to flow over the edges of the molds. For the final step, the material was cured at room temperature for twenty-four hours.

#### 2.2.3. Preparation of Experimental Group Specimens Reinforced with the Chitosan–TiO_2_ Synthesized Nanocomposite

The chitosan–TiO_2_ synthesized nanocomposite powder was added to ethanol in specific weight percentages, with ethanol volumes varying according to the quantity of silicone base used for fabricating five specimens. Absolute ethanol of 99.5 wt.% concentration (EM- PARTA ACS; Merck-KGaA, Darmstadt, Germany) served as the facilitating medium for the dispersion of chitosan–TiO_2_ nanocomposite powder within the silicone base [35]. This blend of ethanol and the chitosan–TiO_2_ synthesized nanocomposite was then subjected to a sonication process (Q700 Sonicator; Qsonica LLC, Newtown, CT, USA) for thirty minutes at room temperature, employing continuous cooling. An amplitude power of 400 W was utilized with a five-second pulse-on time and a two-second pulse-off time. A standard titanium alloy probe (#4220) with 1/2-diameter dimensions of 136 × 13 mm was deployed.

The synthesized chitosan–TiO_2_ nanocomposite with ethanol was combined with the RTV silicone type A-2186 base to prepare specimens for the experimental groups. The mixture was processed in a vacuum mixer for ten minutes. Subsequently, the vacuum mixer’s jar was positioned over a magnetic hotplate stirrer (LabTech LMS-1003; Daihan Labtech Co., Ltd.) and linked to a vacuum rotary pump (EuroVac; Thompson CSF, Sunnyvale, CA, USA). This arrangement facilitated the evaporation of ethanol under a pressure of −0.075 MPa for 120 min. During these two hours, the mixture was stirred every three minutes for one minute to ensure the homogeneous dispersion of the chitosan–TiO_2_ synthesized nanocomposite within the silicone.

The blend was then cooled to room temperature prior to the addition of the catalyst. The mixture was processed for an additional five minutes in the vacuum mixer. It was then poured into molds and moved to a vacuum chamber for two minutes to purge any air bubbles. G-clamps were utilized to secure the molds, allowing excess silicone material to seep over the molds’ edges. The material was left undisturbed to cure at room temperature for 24 h. Finally, the specimens were trimmed and marked to distinguish them between different groups.

#### 2.2.4. Preparation of the Experimental Group Specimens

Specimens for the experimental groups were crafted by integrating one variety of nanoparticles (either TiO_2_, chitosan, or a TiO_2_–chitosan nano-combination (TC)) at concentrations of 2 wt.%, 3 wt.%, and 1 wt.%, respectively, by weight with the silicone base. The mixing procedure was executed in a manner similar to the method used for the control specimens and in compliance with the manufacturer’s instructions.

For each tensile and percentage of elongation, tear, hardness, and surface roughness tests, one hundred and fifty specimens (with an average of five from each silicone category) were evaluated after exposure to five accelerated aging conditions. It is crucial to recognize that all the mechanical properties of the specimens were also evaluated at a baseline value, i.e., twenty-four hours after preparation with no artificial weathering. This was performed for an average of five specimens from each silicone category (baseline group). Figure 1 illustrates the study design.

#### 2.2.5. Conditioning Modes

The specimens were methodically divided into six groups, each signifying a distinct conditioning process, with an additional baseline group. The first process entailed storage in a commercially available antibacterial silicone-cleaning solution (B-200-12, Daro Inc., Lakeside, AZ, USA) for thirty hours. Then, immersion in an artificial sebum solution for six months was performed. This sebum solution was manufactured by dissolving 10 wt.% of palmitic acid with 2 wt.% of glyceryl tripalmitate into 88 wt.% of linoleic acid (all *w*/*w*) [34,37,38,39]. The third procedure involved storage in simulated acidic perspiration, or sweat, for six months. The sweat solution was prepared following the International Organization for Standardization standard ISO 105-E04:96 [40]. It consisted of 0.5 g of L-histidine monohydrochloride monohydrate, 5 g of sodium chloride, and 2.2 g of sodium dihydrogen orthophosphate dehydrate per liter of distilled water [34,37,38,39]. The fourth condition involved the specimens experiencing accelerated artificial ultraviolet (UV) radiation weathering for 720 h. The aging chamber employed for this process was equipped with two light fixtures providing ultraviolet light exposure equivalent to 720 KJ/m^2^/h while maintaining a constant temperature of 60 °C and a relative humidity of 80% [41].

Finally, the specimens were subjected to natural outdoor weather conditions for six months. This exposure was conducted in compliance with the American Society for Testing and Materials Designation G7-8.3.1 [42]. The specimens destined for this process were carefully suspended from stainless steel racks and strategically placed on the rooftop of the College of Dentistry at the University of Sulaimani for six months (June to December 2022) [36,38,42]. The monthly average high and low temperatures and the climate data are recorded in Table 1. The specimens were left open and exposed to the environment throughout the weathering process. They were positioned in a way where no obstructions were evident on any side, guaranteeing maximum exposure to the environmental conditions. These specimens were examined daily to ensure no alteration in their position. Before the evaluation was conducted, they were cleaned for ten minutes in distilled water, wiped dry, and then tested.

These conditioning durations were selected to emulate the usage of silicone prostheses throughout twelve to eighteen months. A typical patient would wear their prosthesis for eight to twelve hours daily, during which it was projected to be exposed to one to three hours of daylight, standard environmental conditions, and continual sebum and sweat when affixed to the affected site. Additionally, a typical cleaning routine of around five minutes was performed prior to bedtime. Therefore, a service duration of one month equated to roughly thirty to ninety [41] hours of daylight aging, ten to fifteen days of immersion in sebum or acidic solutions (sweat), and one hundred and fifty minutes of immersion in cleaning solutions [37,39].

#### 2.2.6. Mechanical Tests

The procedures of this experiment were executed in strict compliance with the standards stipulated by the International Organization for Standardization (ISO) and the American Standards for Testing and Materials (ASTM) pertaining to vulcanized rubber. Before any testing was performed, the specimens were subjected to a 24 h conditioning period at a regulated room temperature of 23 ± 1 °C and a relative humidity of 50 ± 5%. Additionally, a minimum time interval of 16 h was mandated between the vulcanization process and the subsequent testing procedures.

In an effort to uphold the integrity and properties of the specimens, stringent preservation methods were employed. These included storing the samples within hermetically sealed bags and impervious boxes that were resistant to light to mitigate the risk of potential alterations affecting them [37,43].

##### Tensile Strength Test

Tensile tests were conducted in accordance with the ISO 37 (2017) [44] standard, utilizing type-two dumbbell-shaped specimens. Thickness evaluations were performed at three distinct points (both ends and the center) of the specimens with a digital caliper (INGCO, China). The mean value of these three measurements was utilized to perform subsequent computations.

For the execution of the tensile strength (Ts) tests, a Laryee UE34100 Computer Control Electronic Universal Testing Machine was employed. Each specimen was precisely positioned within the tensile-testing clamps, ensuring the end tabs were symmetrically gripped to facilitate an even distribution of tension across the cross-section. The specimens were stretched at a rate of 500 mm/min, and the peak stretching force at the point of rupture (break) was logged using the computer software. Any specimens that fractured outside the designated narrow portion or demonstrated deformation outside the test length were excluded from the evaluation.

The calculation of the ultimate tensile strength involved dividing the maximum force, recorded in newtons (F), by the original cross-sectional area of the specimen, which was determined by the product of the width (W) and thickness (T) of the narrow section of the dumbbell-shaped specimen. The formula used (Equation (1)) for this calculation is as follows:

Ts (MPa) = F(N)/W × T (mm^2^) (1)

where

Ts: ultimate tensile strength in megapascals (MPa);

F: maximum force recorded during the test in newtons;

W: width of the narrow portion of the specimen in millimeters;

T: thickness of the narrow portion of the specimen in millimeters.

##### Elongation Percentage

Pursuant to the procedures established in ISO 37 (2017) [44], the percentage of elongation was tested simultaneously with the measurement of tensile strength prior to the specimen’s point of failure. The initial length of the specimen was assessed before the onset of tensile testing, utilizing a digital caliper (INGCO, China).

For the elongation test, two benchmarks were imprinted on the narrow section of the dumbbell-shaped specimen using a fine-lined permanent marker. These marks were set at a distance of 20 ± 0.5 mm from each other and were evenly spaced from the specimen’s center, perpendicular to its longitudinal axis.

During the tensile test, the specimen eventually reached a point of failure and fractured. The elongation at this point of breakage was evaluated by comparing the initial length of the tensile specimen (L_o_) to its length at the point of breakage (Lb).

The elongation percentage at break was then determined using the following formula (Equation (2)):

Elongation percentage at break = 100 × (Lb − L_o_)/L_o_ (2)

where

L_o_ denotes the initial test length of the specimen, which is 20 mm;

Lb signifies the test length of the specimen at the point of breakage, measured in millimeters.

##### Tear Strength Test

In strict adherence to the ISO 34-1:(2015) [45] standard, the tear strength test was implemented using trouser-shaped test specimens. Each trouser leg of the specimen was symmetrically inserted into the grips and carefully aligned with the direction of the pull. To effectively secure the specimen, it was inserted to a depth of 30 mm within the grips. The Laryee UE34100 Computer Control Electronic Universal Testing Machine, a computer-operated universal testing apparatus, was utilized for the test. Throughout the testing process, the specimens were subjected to a consistent strain rate of 500 mm/min.

To assess the tear strength, expressed as newtons per millimeter of thickness (N/mm), the following formula (Equation (3)) was applied:

Tear strength (T) = F/d (3)

where

T represents the tear strength in newtons per millimeter (N/mm);

F signifies the maximum force recorded during the test, measured in newtons (N);

d denotes the thickness of the test specimens in millimeters (mm).

##### Hardness Test

The indentation hardness test was performed diligently in alignment with the ASTM D224015 (2021) [46] standard. The specimens were prepared as 40 × 40 × 6 mm squares, each marked at points distanced 12 mm from any edge and spaced 6 mm apart from the nearest point. The Shore A Durometer Tester Meter (Model: LX-A; total measure force: 10 N; needle stroke: 2.5 mm; needlepoint size: Φ0.79 mm; resolution: 0.5 HA; dial value: 0~100 HA; recommended range of measurements: 10~90 HA, B089YDB9LL Co, China Ltd.) was utilized to perform the testing procedure.

The instrument was positioned vertically in this process, with the presser foot aligned parallel to the specimen’s surface. Readings were noted on the specimen’s surface after a contact period of five seconds. Six indentation points were pre-marked on each specimen, and the average reading from these points was obtained to calculate the specimen’s overall hardness value.

##### Surface Roughness

The surface roughness was evaluated for specimens prepared for Shore A hardness testing, each measuring 40 mm × 40 mm × 6 mm. The instrument employed for this assessment was a surface roughness tester (TR200, INNOVATEST Europe BV, Borgharenweg 140, Maastricht, The Netherlands); for the comprehensive analysis, the mean value of six readings was computed.

To precisely gauge the minor irregularities on the texture of the specimen’s surface, a portable digital surface roughness tester was utilized. Each specimen was meticulously positioned on a stable and rigid surface. Then, six measurements were performed on each sample, which involved tracing the specimen’s surface at six distinct points using a diamond stylus integrated within the tester. The mean value of these readings was subsequently determined, providing an accurate measure of the surface’s average roughness value.

### 2.3. Statistical Analysis

A comprehensive statistical analysis was performed to succinctly encapsulate this study’s outcomes for every inspected variable. For continuous variables, the mean and standard deviation were presented. The data related to each attribute were subjected to Levene’s test for homogeneity of variance (α = 0.05) to ascertain if equal variances were permissible (*p* > 0.05). If this was the case, one-way ANOVA and Tukey’s HSD tests were performed to scrutinize significant disparities between the test groups within properties and specimens.

If equal variances could not be assumed, Dunnett’s T3, a test for multiple comparisons, was used to examine any notable differences between the test groups (*p* < 0.05). A *p*-value of 0.05 or less was considered statistically meaningful across all the conducted tests. Shapiro–Wilk and Kolmogorov–Smirnov tests were employed to ensure the variables in this analysis adhered to a normal distribution, which validated the use of the one-way ANOVA test. These statistical analyses were conducted using the SPSS program for Windows, version 27.0.

## 3. Results

### 3.1. Morphological Characteristics

#### 3.1.1. Field Emission Scanning Electron Microscopy

**Figure 2:**
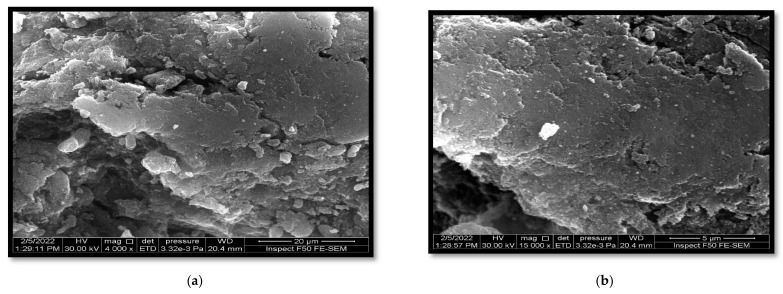

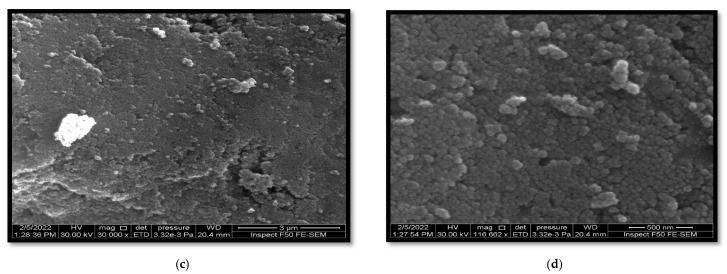
FESEM of the synthesized nano-composite chitosan–TiO_2_. (**a**) At a 20 µm resolution. (**b**) At a 5 µm resolution. (**c**) At a 3 µm resolution. (**d**) At a 500 nm resolution.

Field emission scanning electron microscopy (FESEM) was employed to investigate the surface morphology of various nanoparticle coatings and to obtain insights into the external morphology of the nanocomposite under study. The outcomes of this study show that FESEM plays a pivotal role in verifying the even and homogenous dispersion of nanosized, minimally aggregated TiO_2_ particles within the chitosan (CS) matrix, as illustrated in Figure 2.

The observed irregular spherical morphologies can be attributed to the interlocking interaction between TiO_2_ and CS, which subsequently enhances the homogeneity of the two components.

#### 3.1.2. X-ray Diffraction (XRD)

**Figure 3:**
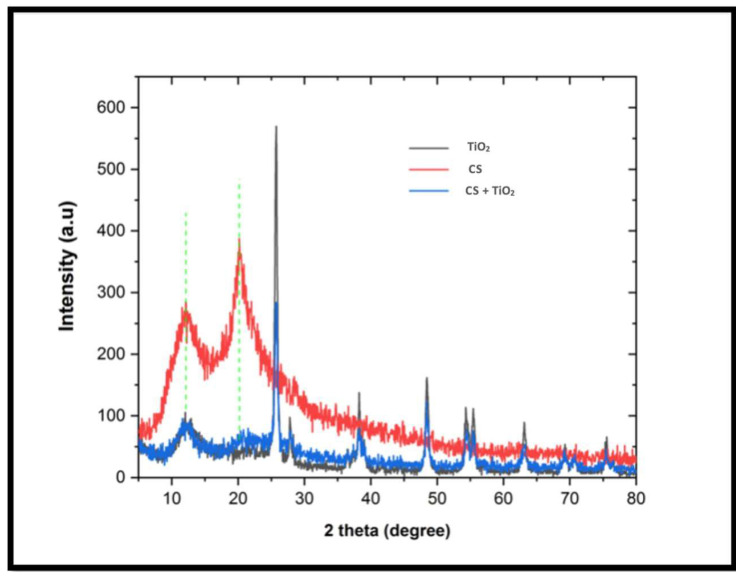
X-ray diffraction patterns of TiO_2_, chitosan, and chitosan–TiO_2_.

The crystalline structure of the synthesized nanocomposites was analyzed by using the method of XRD. The XRD patterns of the synthesized chitosan–TiO_2_ nanocomposite, pure TiO_2_, and chitosan are presented in Figure 3. The XRD pattern of chitosan displayed small, broad peaks at (11.8°) and (20.0°), indicating its semi-crystalline structure. This suggests that the incorporation of TiO_2_ into the chitosan matrix predominantly occurs in the semi-crystalline region of chitosan.

In contrast, both chitosan and TiO_2_ exhibited distinct diffraction peaks in the XRD pattern of the nanocomposite. The TiO_2_ nanoparticles displayed the presence of anatase and rutile forms. The coexistence of these mixed phases of TiO_2_ was advantageous in minimizing the recombination of photogenerated electrons and holes, thereby enhancing the photocatalytic activity of titanium.

#### 3.1.3. Fourier Transform Infrared Spectroscopy (FTIR)

**Figure 4:**
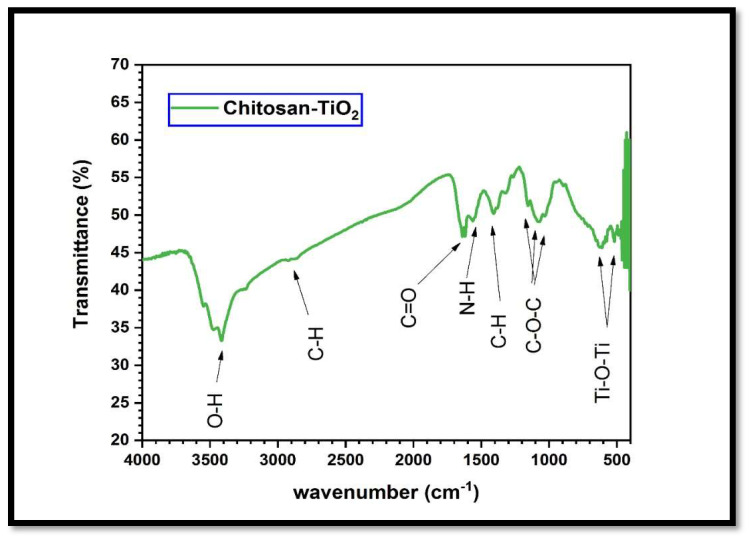
FTIR of the synthesized chitosan–TiO_2_ nanocomposite.

The FTIR analysis presented in Figure 4 reveals the presence of characteristic bands corresponding to the chitosan–TiO_2_ nanocomposite, which provides a greater insight into the synthesized nanocomposite of chitosan–TiO_2_. In the FTIR spectra, it can be observed that the peaks around (3441, 3437), (2855, 2926), and (1621, 1619) cm^−1^ are related to (O–H), (C–H), and (C=O) groups, respectively. The peaks around (1575, 1500), (1425, 1352), and (1112, 1040) cm^−1^ are related to (N–H), (CH–OH), and (CH2-OH) groups, respectively. The fingerprint band between 700 and 400 cm^−1^ shows stretching vibrations of (Ti-O-Ti), which indicates the immobilization of TiO_2_ onto the chitosan matrix.

### 3.2. Results of the Mechanical Tests

#### 3.2.1. Tensile Strength Test Results

The data for the tensile strength averages and standard deviations for all the samples are presented in Table 2. Significant variances in the tensile strength characteristics were observed for the silicone specimens subjected to sweat, UV, and outdoor weathering conditions, as evidenced by the *p*-values of 0.05, 0.000, and 0.005, respectively.

Specifically, when tested under various conditions, the 1 wt.% TC specimen’s tensile strength was significantly changed (*p*-value: 0.01). Outdoor weathering resulted in the most considerable increase in tensile strength (11.29 ± 0.85) for the 1 wt.% chitosan–TiO_2_ sample (*p* < 0.05).

The 1 wt.% chitosan–TiO_2_ specimen, when exposed to sweat for six months, exhibited a tensile strength (10.23 ± 0.70) that was statistically greater than that of the 1 wt.% TC specimen (*p* < 0.05). Similarly, under the UV weathering condition, the 1 wt.% chitosan–TiO_2_ specimen presented a significantly superior tensile strength (11.29 ± 1.01) compared to all other specimens (*p* < 0.05).

Interestingly, the 1 wt.% TC specimen demonstrated a significant increase in tensile strength values when tested under sebum conditions compared to those in the baseline condition (*p* < 0.05).

#### 3.2.2. Elongation Percentage Results

The average elongation percentages and their corresponding standard deviations for all test specimens are outlined in Table 3. There are notable differences in the elongation percentage among the silicone specimens across all conditions and within the control (zero nano) specimen under varying conditions. When subjected to sweat for six months, the 1 wt.% chitosan–TiO_2_ and 3 wt.% chitosan specimens showed the lowest elongation values of 373.21 ± 35.97 and 377.32 ± 42.47, respectively, which were statistically significant (*p* < 0.05).

In comparison to the control (zero nano) specimen exposed to sebum for six months, the 3 wt.% chitosan specimen exhibited a significantly reduced elongation percentage (*p* < 0.05).

When the specimens were exposed to antibacterial conditions for thirty hours, the 1 wt.% chitosan–TiO_2_ specimen showed a significantly lower elongation percentage (*p* < 0.05) compared to the 1 wt.% TC, 2 wt.% TiO_2_, and control (zero nano) specimens.

However, when exposed to sweat, sebum, and outdoor weathering for six months, the control (zero nano) specimen displayed significantly reduced elongation percentages (*p* < 0.05) compared to its baseline condition.

#### 3.2.3. Tear Strength Test Results

The mean values and corresponding standard deviations for the tear strength test of all specimens are detailed in Table 4. Significant variations in the tear strength amongst the silicone specimens under baseline and sweat conditions were observed.

When subjected to diverse conditions, the tear strength of the 1 wt.% chitosan–TiO_2_ and 3 wt.% chitosan specimens varied significantly, with *p*-values of 0.02 and 0.000, respectively. Sweat exposure led to the lowest tear strength value (26.67 ± 2.08) for the 3 wt.% chitosan specimen, significant at the *p* < 0.05 level.

Upon exposure to sweat for six months, the tear strength value (34.22 ± 5.18) of the 2 wt.% TiO_2_ specimen was considerably higher (*p* < 0.05) than that of the 3 wt.% chitosan specimen. Similarly, under outdoor weathering conditions, the 1 wt.% chitosan–TiO_2_ specimen demonstrated a significantly elevated tear strength value (34.44 ± 2.36) compared to the 3 wt.% chitosan specimen (*p* < 0.05). However, under sebum conditions, the tear strength of the 3 wt.% chitosan specimen was significantly higher (*p* < 0.05) than its corresponding baseline condition. The 1 wt.% chitosan–TiO_2_ specimen, when exposed to outdoor weathering and antibacterial conditions, exhibited significantly increased tear strength values (*p* < 0.05) compared to the 1 wt.% chitosan–TiO_2_ specimen under baseline conditions.

#### 3.2.4. Hardness Test Results

The mean values and standard deviations of the hardness test for all examined samples are displayed in Table 5. The hardness values among the silicone categories were significant in all conditions, except under baseline conditions.

When exposed to different conditions, significant variations were observed in the hardness values of 2 wt.% TiO_2_, 3 wt.% chitosan, and the control (zero nano) specimens, as substantiated by *p*-values of 0.001, 0.03, and 0.001, respectively. The smallest hardness value (35.96 ± 1.18), significant at the *p* < 0.05 level, was found in the 1 wt.% TC specimen after six months of sebum exposure.

A significant reduction in the hardness value was seen in the 1 wt.% TC specimen compared to the 3 wt.% chitosan specimen when subjected to six months of sweat exposure. Similarly, when exposed to outdoor weathering, the 1 wt.% chitosan–TiO_2_ specimen displayed a significantly reduced hardness value compared to the 3 wt.% chitosan specimen.

Conversely, the 2 wt.% TiO_2_ silicone category exhibited significantly elevated hardness values under all conditions, except sweat exposure, compared to the baseline condition of the same silicone category. Similarly, the hardness values of the 3 wt.% chitosan specimen increased significantly under sweat, UV, and outdoor weathering conditions compared to its baseline condition. All conditions in the control (zero nano) specimen led to significantly increased hardness values compared to its baseline condition.

#### 3.2.5. Surface Roughness Test Results

The mean values and standard deviations for the surface roughness attributes of all the evaluated specimens are outlined in Table 6. A marked distinction in surface roughness properties among the silicone specimens was observed across all conditions.

All specimens exhibited significant variations in surface roughness values when exposed to differing conditions, denoted by a *p*-value of 0.000. One month of UV artificial weathering produced the most minor surface roughness value (0.20 ± 0.0007) for the 1 wt.% TC specimen, deemed significant at the *p* < 0.05 level.

When exposed to six months of sweat and thirty hours of antibacterial treatment, the surface roughness of the 1 wt.% TC specimen was significantly lower than that of all other specimens. The 2 wt.% TiO_2_ specimen also demonstrated a significantly lower surface roughness compared to all other specimens when subjected to outdoor weathering.

On the contrary, the surface roughness values of the 1 wt.% chitosan–TiO_2_ specimen were significantly higher in all conditions (except six months of outdoor weathering) compared to its baseline condition. Conversely, the 3 wt.% chitosan specimen showed significantly lower surface roughness values across all conditions compared to its baseline condition. Likewise, the 1 wt.% TC and control (zero nano) specimens, when exposed to all conditions and excluding six months of outdoor weathering, showed significantly reduced surface roughness values compared to their respective baseline conditions.

## 4. Discussion

A pioneering methodology was developed which synthesized a unique three-phase composite by impregnating nanoparticles with a polymeric silicone, an endeavor not previously undertaken. The composite was meticulously fabricated by adeptly incorporating two distinct particulates—nanoparticles (TiO_2_) and microparticles (chitosan)—in well-defined ratios. This integration led to notable enhancements in the inherent properties of silicone polymers. This avant-garde progress marks a significant leap towards refining maxillofacial prosthetics, endowing them with unparalleled attributes and realism. The intrigue surrounding hybrid nanoparticles, specifically CS–TiO_2_ assemblies, is justified, as they harmoniously combine the attributes of organic and inorganic constituents, culminating in materials with heightened and distinct characteristics [19,47].

Core–shell nanoparticles, encapsulating one material within another, present superior biocompatibility and enhanced stability as compared to traditional nanoparticles. Their specialized surface properties enhance their role in biomedical contexts, particularly for drug delivery. Concurrently, the diverse applications of the CS–TiO_2_ hybrid composite range from antimicrobial actions to environmental pollutant breakdown. Given these advancements, the CS–TiO_2_ core–shell nanocomposite showcases promising potential across medical and technological arenas [47,48].

In this investigation, the core–shell combination technique was proficiently utilized to fabricate a TiO_2_-supported chitosan nanocomposite. Rigorous morphological characterizations, encompassing scanning electron microscopy (SEM) (depicted in Figure 2), X-ray diffraction (XRD) (elucidated in Figure 3), and Fourier transform infrared spectroscopy (FTIR) (presented in Figure 4), unequivocally ascertained the effective integration and homogeneous dispersion of the TiO_2_ nanopowder within the chitosan matrix. These analyses highlight the precise synthesis and interlacement of TiO_2_ and chitosan constituents within the nanocomposite structure.

Ensuring an even dispersion of chitosan–TiO_2_ nanopowder within a silicone matrix remains arduous due to inherent nanoparticle agglomeration, though it is imperative for enhancing mechanical properties in polymer/nanocomposites. Ethanol, characterized by its polar nature and hydroxyl (OH) groups, affords sustained dispersion capabilities [49].

In this study, an innovative approach was adopted where the nanocomposite powder was subjected to sonication in ethanol, subsequently integrated with silicone, and then processed via heat and vacuum techniques to expel the ethanol, ensuring a superior dispersion without necessitating auxiliary materials that might adulterate silicone attributes [35].

Within the expert communities of anaplastologists, maxillofacial prosthodontists, and dental technicians who focus on crafting facial prostheses, RTV silicone elastomers are the preferred material [50]. The additional polymerization RTV silicones possess enhanced mechanical properties, simplifying mold design and management while facilitating internal and external pigmentation. When contrasted with other silicones, these retain their hue more effectively and are biocompatible [20].

The observed variations in the silicone elastomer materials’ physical and mechanical characteristics stem from the distinct ingredients in their formulations. This encompasses diverse crosslinking approaches (such as addition or condensation), variations in the molecular weight of polydimethylsiloxane (PDMS), inconsistencies in crosslink density, and the specific grade and concentration of the silica filler in the matrix [4].

The extent of crosslinking is influenced by factors such as the type and concentration of the thermal initiator, fillers, additives, curing temperature, and the duration of polymerization. The optimal blend of elastomer and colorant should not just provide pleasing esthetics in a clinical setting. Moreover, it should preserve these esthetics and physical attributes for a prolonged period, or at least until changes in the patient’s tissues affect the prosthesis fit [51,52].

A-2186 might exhibit its characteristics because of its increased filler content or the higher molecular weight of its dimethylsiloxane polymer. This elastomer, A-2186, is a two-component silicone elastomer that vulcanizes at low temperatures through an additional crosslinking process. The curing method for A-2186 involves crosslinking polysiloxanes. This is achieved by adding a group containing silyl hydride (–SiH) to a vinyl group (CH=CH_2_) associated with silicone, facilitated with a platinum catalyst [53,54].

The crosslinking process initiates by forming a bond between the vinyl group’s double bond and the platinum. Subsequently, this reacts with the silyl hydride group. Any chemical compound that can compete with this double bond during the formation of the platinum–vinyl complex might hinder the hydrosilylation crosslinking process. Therefore, organic compounds containing elements with a lone pair of electrons, such as amines, sulfites, or phosphines, have the potential to form a bond with the platinum, disrupting the crosslinking process. It is worth mentioning that the curing of elastomers similar to A-2186 can be affected by even minute amounts of substances, such as amines, sulfur, organo-tin compounds, nitrogen oxide, and carbon monoxide [55].

A-2186 silicone is distinguished among its counterparts for its foundational color-packing capabilities. Its superior tensile strength and pronounced resistance to tearing augments the facial prostheses’ edge quality, rendering them more resilient and less vulnerable to degradation. Moreover, compared to its counterparts, A-2186 manifests a tactile softness reminiscent of authentic human skin, enhancing its aesthetic sophistication [32,50,54,56].

Relevant mechanical properties integral to the efficacy of maxillofacial prostheses include aspects such as hardness, tensile strength, tear strength, and elongation [55].

Upon the precise analysis of this research, it is evident that the integration of the synthesized hybrid chitosan–TiO_2_ nanocomposite markedly influences the mechanical and physical characteristics of the maxillofacial A-2186 silicone elastomer. This influence remains pronounced even under a spectrum of accelerated aging conditions. Given these observations, the null hypothesis was rejected.

### 4.1. Aging Conditions

Factors leading to the degradation of outdoor silicone polymers include exposure to sunlight, moisture, and varying temperatures. The extent of these changes can differ based on the geographical area, climate, and the specific environment in which a prosthesis is used. An accelerated aging chamber is utilized to assess the durability of maxillofacial materials. This chamber subjects samples to radiation, heat, and humidity resembling atmospheric conditions. However, this method does not perfectly replicate the actual conditions faced by patients. The rapid aging process in the chamber can alter the degradation mechanism of the polymer, resulting in imprecise assessments of its color stability [57,58].

In this study, five unique accelerated aging conditions were implemented to best represent the real-life situations encountered by a patient. The prosthesis is positioned on a defect site, exposing it to body secretions, such as sweat and sebum. The prosthesis undergoes regular cleaning and is subjected to outdoor environmental challenges. Additionally, accelerated UV artificial aging was introduced to determine its impact on the properties of the silicone.

Weathering can induce significant alterations in the mechanical and physical properties of polymers, as illustrated by Eleni et al. [59] and Nobrega et al. [60]; when subjected to photo-oxidative degradation, the ensuing mechanisms can be delineated as follows:

**Initiation Phase:** This entails the genesis of free radicals. Such formations can be attributed to the photon-induced dissociation of a polymer molecule or due to contaminants, like trace metals originating from the polymerization catalyst.

**Propagation Phase:** At this juncture, the extant polymer radicals engage with oxygen, leading to the emergence of polymer oxy- and peroxy-radicals and consequential secondary polymer radicals. This invariably culminates in the chain scission of the polymer.

**Termination Phase:** During this phase, disparate free radicals undergo reactions with one another, potentially resulting in augmented crosslinking within the polymer matrix.

Irradiated polymers experience pivotal structural shifts. Notably, there are variations in their molecular weight distribution because of chain scission, crosslinking, and end-linking activities. Additionally, there is a manifestation of volatile degradation byproducts. Such modifications invariably impact the inherent physical characteristics of the polymer. The repercussions on the polymer’s structural framework are multifaceted. During crosslinking, there is a noticeable increase in the density of the structural network due to the formation of inter-monomeric or inter-chain bonds, rendering the polymer more rigid. In contrast, when chain scission predominates, the structural network’s density diminishes, making the polymer more malleable. Both phenomena are quintessentially observed in polymers subjected to irradiation [59,60,61].

Polymers commonly possess aromatic rings and C=C bonds within their structural makeup. These constituents are proactive to absorb ultraviolet rays, especially during accelerated aging conditions. Based on studies by Goiato et al. [62] and Nobrega et al. [32], molecular destabilization in polymer molecules has been suggested upon the absorption of UV light. Interestingly, the capability for the surplus energy from this destabilization to be conveyed from one molecule to another exists, allowing the stability of the primary molecule to be re-established. Once this energy transition has occurred, longer wavelengths, such as visible light or even heat, may manifest. However, a process known as photochemical degradation can be initiated by releasing this energy, leading to the inevitable degradation of the molecular structure. Furthermore, this cascade of events might provide insights into the dimensional modifications observed in silicone materials, as proposed by Goiato et al. [15]. Aging also leads to a decreased surface roughness, resulting in a smoother surface over time [63,64].

Additionally, Mouzakis et al. elucidated that many polymers undergo distinct molecular transformations upon exposure to ultraviolet radiation (UV). Manifestations of these transformations include the cleavage of primary chains and the generation of radicals, amongst other molecular deviations. Consequent to these alterations, one often observes changes in the mechanical characteristics of the material. Notably, post-UV exposure, there is a discernible increase in the intrinsic crystallinity of the material. A conceivable hypothesis for this development posits that UV-induced disruptions within the amorphous regions of the polymer lead to the formation of novel, remarkably pliable chain segments. These newly formed segments subsequently tend to orient themselves into more regimented configurations, a phenomenon aptly described as chemical crystallization, leading to heightened polymer stiffness [65].

Crosslinking, which forms between chains or existing monomers, heightens density, subsequently boosting the tensile strength and the extent of the silicone stretch. In contrast, chain fragmentation results in bond disruptions, either internally or interchain, weakening the overall polymer structure and causing reductions in both tensile strength and the ability to elongate. The aging observed in silicone rubbers is mainly due to the chemical deterioration of connectors binding polysiloxane chains and the methyl groups affixed to silicone elements [66,67].

### 4.2. Tensile Strength

A silicone elastomer’s tensile strength delineates the material’s overarching durability, and the resultant elongation provides insights into the pliability of a prosthesis. Tensile strength characterizes the utmost stress a material can tolerate before undergoing localized rapid deformation. Such a trait is vital for maxillofacial silicone. Pronounced tensile forces are exerted on more slender segments of the material, particularly when a patient extracts a prosthesis [4,68].

Based on this study’s outcome, exposure to sweat, UV artificial weathering, and natural outdoor weathering significantly (*p* < 0.05) impact the tensile strength of all silicone categories. These external factors, especially UV-induced photochemical aging, alter the physical and mechanical properties of the silicone polymer, leading to increased molecular crosslinking and enhanced silicone density [59,60]. The tensile strength and elongation of silicone are heavily influenced by the crosslinking between the silicone chains. Specifically, in the context of this study, the A-2186 silicone demonstrated an enhanced tensile strength following such exposures. Such amplification can be attributed to its intrinsic composition, where the A2186 silicone may inherently possess a higher filler loading and/or higher molecular weight within its dimethylsiloxane polymer, bolstering its resilience against aging processes. This observation is consistent with the research conducted by Dootz et al. [56], which highlighted that the tensile strength of A-2186 silicone withstood the effects of accelerated aging and surpassed the strength of other evaluated materials.

Moreover, this research elucidated that the synergistic incorporation of two discrete nanoparticles, namely TiO_2_ and chitosan, into the silicone matrix augments its tensile strength. Specifically, silicone specimens infused with 1 wt.% chitosan–TiO_2_ exhibited enhanced tensile strength when subjected to conditions such as UV artificial weathering, outdoor weathering, and sweat. This observation aligns with the findings of Hatamleh et al. [36], who observed that the tensile modulus experienced a significant boost with a 1 wt.% additive nanoparticle concentration. Gandhi and Sethuraman [20] highlighted that integrating 1 wt.% nanoparticles into RTV silicone markedly improved features such as tear and tensile strength, hardness, and color stability compared to traditional silicone. This amplified performance was especially noticeable after the material was subjected to six months of simulated accelerated aging.

Research by Han et al. [30], Wang et al. [10], and Bangera and Guttal [9] emphasized the critical importance of embedding nanoparticles into silicone. This integration has consistently been tied to enhancing the silicone’s mechanical facets. An exciting feature of nanoparticles is their role as UV protectants. Their diminutive size, smaller than the wavelength of UV light, allows them to effectively shield against these rays. When subjected to UV radiation, the electrons within the nanoparticles vibrate, dissipating and absorbing portions of the light.

Specifically, Han et al. [30] highlighted that including nano-TiO_2_, especially at a concentration of 2.0 wt.% by weight, elevates the mechanical properties of materials, likely due to its vast specific surface area, which increases its engagement with the surrounding environment [22]. Meanwhile, Wang et al. [10] emphasized that dispersing these nanoparticles uniformly within the silicone matrix not only increases its structural strength but also promotes the formation of a crosslinked structure, as this dispersion likely facilitates an expansion of the cross-sectional area, intensifies the force, and fosters the establishment of a crosslinked configuration in the composite material. Lastly, Bangera’s [9] findings indicate that such nanoparticles reinforce the material’s strength, provide resilience against potential environmental stresses and aging, and simultaneously refine its optical and physical characteristics.

Additionally, Andreopoulos and Evangelatou [69] and Radey et al. [34] observed that, when subjected to tensile forces, the polymer chains and nanoparticles glide across one another. The presence of these nanoparticles significantly aids in guarding the polymer chains from potential fractures. This is further accentuated by TiO_2_, which acts as a comprehensive crosslinker, forming robust hydrogen bonds between its surface and the poly(dimethylsiloxane) chains. As a result, the polymer gains increased rigidity and strength due to the augmented crosslinking density. These crosslinks act as a defensive mechanism when under tension, preventing the poly(dimethylsiloxane) chains from breaking and enhancing tensile strength.

In contrast to the findings of this research, the study by Ibrahim and Al-Judy [31] revealed a significant decrease in the tensile strength of silicone elastomers with the addition of chitosan, with the lowest strength observed at 3.5 wt.% chitosan. The disparity in the results might stem from variations in the methodology and the specific type of RTV silicone used. Although both studies sourced their silicone from the same company, viscosity and inherent tensile strength differences could have influenced the outcomes. Moreover, this study opted for microparticle, low-molecular-weight chitosan, incorporated at 3 wt.% by weight into the silicone. This choice was made to minimize chitosan particle aggregation, a challenge that may have arisen in Ibrahim and Al-Judy’s [31] study.

Additionally, the data revealed an enhancement in tensile strength under sweat conditions, specifically in the 1 wt.% chitosan–TiO_2_ silicone category, which may be attributed to the acidic nature of sweat potentially serving as a catalytic agent, expediting the polymerization process and crosslinking. This could also be influenced by the interaction between TiO_2_ nanoparticles and the chemical components of sweat [38]. Notably, finer nanoparticles tend to have a more profound interaction with the silicone’s polymeric chain [70]. These insights align with Radey et al.’s [34] research. However, in contrast, Hatamelh et al. [37] documented disparate effects when examining the impact of sweat on a different silicone elastomer variant, one devoid of nanoparticle incorporation. In their examination, sweat instigated the deterioration of the silicone’s polymer network junctions, resulting in their susceptibility to fracture under reduced force applications.

Furthermore, the 1 wt.% TC sample showed a significant increase in tensile strength under sebum conditions compared to its baseline condition. This might be due to the combined effects of TiO_2_ and chitosan interacting with the silicone, possibly leading to intensified crosslinking or a denser structure. Conversely, Hatameleh et al.’s study [36] found that the fatty acids in sebum can weaken and soften silicone by breaking down its polymer chains.

### 4.3. Elongation Percentage

Elongation, which gauges malleability, is crucial in determining a facial prosthetic elastomer’s ability to withstand rupture throughout its use. This metric is especially vital when considering the removal of nasal or ocular prostheses. The resulting elongation reflects the material’s flexibility, mirroring how it aligns with natural facial motions. It also signifies the material’s resilience to breaking during regular use and maintenance [4,71].

This research observed a uniform decline in elongation percentages across all silicone categories when subjected to accelerated aging conditions. Such a decrease can likely be ascribed to the crosslinking of polymer chains during aging, since a polymer’s elasticity and strength correlate closely with its molecular weight and crosslinking extent [59]. Interestingly, when nanoparticles were integrated into silicone, they presented a marked protective effect against aging, as evidenced by the improved performance of these specimens compared to those devoid of nanoparticles. This observation resonates with the findings of Hatamleh et al. [37], who reported a reduction in silicone elongation following accelerated aging exposure. Similarly, both Andreopoulos and Evangelatou [69] and Azeez et al. [72] emphasized that the diminished elongation might result from increased polymer rigidity and augmented crosslinking density after nanoparticle inclusion. These nanoparticles foster multifaceted crosslinks and ensnared entanglements, which constrain and limit the mobility of the polymer chains, diminishing their capacity to stretch.

### 4.4. Tear Strength

In clinical practice, the tear resistance of poly(dimethylsiloxane) (PDMS) maxillofacial materials is of paramount importance. During fabrication, adjustment, and removal, multidirectional tear stress may compromise the durability of a prosthesis [73]. The integrity of the material is essential, particularly in sensitive areas adjacent to the eyes and nose. These slender peripheries are meticulously designed to merge the prosthesis into the patient’s inherent facial contours. Ordinarily affixed using specialized medical adhesives, these fine margins are vulnerable to potential tearing during routine procedures, such as nocturnal removal or cleansing. Once torn, the prosthesis is irrevocably damaged, necessitating a replacement. As such, it is imperative to use robust, tear-resistant materials for crafting these prosthetics [4].

This study detected a marked augmentation in tear strength across almost all silicone categories. Such an elevation is a consequence of the intricate crosslinking amongst polymer chains facilitated by ongoing polymerization reactions during the aging process.

Mainly, the silicone variant RTV A-2186 exhibited exceptional tear resistance. This can be ascribed to its relatively viscous consistency upon loading compared to other silicone variants. Also, the advanced mechanical integrity of the A-2186 variant can be attributed either to its pronounced filler composition or to the higher molecular weight inherent to its dimethylsiloxane polymer [54,55,61].

Several studies, such as those by Wang et al. [10], Nobrega et al. [32], Azeez et al. [72], and Sonanahlli et al. [22], have shown that introducing nanoparticles significantly improves the mechanical qualities of silicone, especially its tear strength. Wang et al. [10] argued that this enhancement results from nanoparticle dispersion within the silicone elastomer, expanding its cross-sectional area, boosting force, and creating a composite with a crosslinked structure. According to Nobrega et al. [32], the presence of nanoparticles augments the material’s plasticizing effect, softening it while boosting its resistance to tearing. Azeez et al. [72] pointed out that the filler’s close association with the silicone matrix strengthens its connection to the polymer chains, increasing tear strength. Tear resistance in a material is often linked to the polymer’s ability to distribute energy at the point of a tear’s progression. Other minor fillers help to absorb strain energy in the polymer matrix, enhancing its resistance to tearing and requiring more force to break the chains fully. Sonnahlli et al. [22] suggested that this interaction allows for the better movement of polymer chains around filler particles, strengthening the bond between neighboring PDMS chains. Additionally, these nanoparticles’ inherent surface energy and reactivity promote stronger interactions with the silicone elastomer matrix, crafting a dense three-dimensional framework within the silicone’s structure, as detailed by Goiato et al. [63].

In contrast, Nguyen et al.’s [56] study indicated that including nanoparticles such as TiO_2_ and ZnO does not provide mechanical benefits to silicone elastomers when they are subjected to accelerated aging tests.

In the findings obtained in this research, it was observed that the tear resistance of the silicone category containing 1 wt.% chitosan–TiO_2_ was enhanced when it was exposed to outdoor weathering and antibacterial solution aging conditions. It is believed that this enhancement might be due to the combined effect of chitosan and the TiO_2_ nanocomposite. Consistency with these observations was found in Cevik et al.’s [74] study, where it was posited that the mechanical properties and viscosity of silicone elastomers were improved when nanosized particles were introduced. The crosslinked matrix is believed to be supported by these fillers through their diffusion within it.

However, the detrimental effects of aging conditions on silicone were highlighted, as evidenced by Hatameleh et al.’s [37] study. Their findings demonstrated that, when silicone was exposed to antibacterial solutions, the cleaning solution decomposed into carbon monoxide, carbon dioxide, and sulfur dioxide. The tear resistance of the silicone elastomer might be negatively affected by these byproducts, primarily since the curing process of specific silicone elastomers, comparable to MDX4-4210, is known to be inhibited by substances such as carbon monoxide and nitrogen oxide. Concerning these findings, potential alterations in its physical properties are evident when silicone interacts with such byproducts: the material may exhibit changes in hardness or softness. While profound alterations within the bulk of the materials were not identified, it was mainly on their surface that these modifications became evident post-disinfection with selected antimicrobial solutions, a fact corroborated by Eleni et al. [75]. In conjunction, a reduction in the tear strength of A-2186, when it was subjected to hot and humid outdoor environments, was reported by Al-Harbi et al. [76].

It should be noted that, in the two previous studies, pure silicone was used without the inclusion of nanoparticles. Consequently, this study established that the durability of silicone, when exposed to aging conditions, can be strengthened with the addition of nanoparticles.

On the contrary, the 3 wt.% chitosan silicone category exhibited a decrease in tear strength following a six-month exposure to sweat. This decrease could be attributed to the interaction between chitosan microparticles and the components of sweat, which might be influenced by the inherent acidic disposition of sweat. It is worth noting that chitosan, characterized as a mild base, exhibits insolubility in both aqueous and organic solvents. Nevertheless, it demonstrates solubility in dilute acidic aqueous solutions, mainly when the pH is below six and a half [77].

Furthermore, the diminished tear strength can be ascribed to the inadequate adhesion between the chitosan microparticles and the silicone matrix, consequently heightening the polymer’s vulnerability to tearing. Upon the onset of a tear, the propagation is not limited to the immediate point of origin but is rather accentuated by the micro-defects at the juncture of chitosan and silicone. As chitosan integrates with silicone, these microparticles become encompassed within the elastomer, resulting in a more polished and smoother surface, reducing the likelihood of tearing. Notably, the chitosan augment offers a reinforcing effect: microcracks at the boundary between the silicone and the chitosan play a role in energy absorption. However, an excessive concentration of chitosan leads to its agglomeration, transitioning these microcracks into macro-defects, and thus compromising the tear resistance of the silicone. Such observations align with the findings presented in the studies by both Ibrahim and Al-Judy [31] and Liu et al. [78].

In line with the findings of Sonnahalli and Chowdhary [79], there was a notable decrease in the tear strength at a concentration of 3.0 wt.% of nanoparticles. This might be the case because nanoparticles, at elevated concentrations, tend to agglomerate. Such agglomeration of nanoparticles can exceed the genuine size of the polymer particles, leading to gaps or voids around this agglomerate, which can negatively impact the material’s mechanical properties. When subjected to external forces, these agglomerated particles serve as stress concentration points, diminishing the material’s mechanical strength [30].

Conversely, the presence of sebum enhanced the tear strength of the 3 wt.% chitosan silicone group. The fatty acids in sebum engage in interactions with both silicone and chitosan microparticles, promoting enriched crosslinking within the polymer chains. This enhanced linkage results in a more cohesive molecular arrangement, diminishing the spaces between the chitosan particles and the polymer matrix. Consequently, a more compact elastomeric structure emerges, exhibiting heightened resistance to tearing. Eleni et al. [80] highlighted the potential interactions between fatty acid solutions and the surfaces of the PDMS samples. As a result, there was a noticeable rise in elasticity. This could imply that specific compounds are extracted from the sample matrix when they are subjected to fatty acid solutions. Polyzois et al. [38] further mentioned that the polymer matrix of the PDMS appears to have a better compatibility with such fatty acid solutions.

### 4.5. Hardness

The silicone’s consistency should align with the skin texture of the anatomical region needing restoration. The material’s hardness plays an essential role in determining its texture. The skin over the maxilla’s orbital, nasal, and ear regions is thin and closely adhered to the underlying bone and cartilage. Therefore, for the silicone to closely resemble the texture of these specific areas, its hardness should range between 25 and 35 Shore A [79]. The hardness of maxillofacial materials serves as an index of their adaptability and flexibility. It is imperative that these materials possess a hardness that closely approximates that of the missing facial tissue [4]. Consequently, the hardness of silicone elastomers is determined by the surface properties of the polymer matrix and the density of crosslinks [37,38,80]. Additionally, variations may arise from disparities in the structural integrity of PDMS chains, which are attributed to differences in crosslinking densities and the nature of conditioning [37].

Based on the outcomes of this study, the hardness values exhibited an increase in all silicone categories after being subjected to different accelerated aging conditions when compared to their baseline conditions, except for 1 wt.% TC silicone, which showed a significant decrease in hardness values after six months of immersion in sebum. The pronounced reduction in hardness could potentially arise from the interactions of the sebum fatty acids with the silicone specimen surfaces, as elucidated by Hatameleh et al. [37], Polyzois et al. [38], and Eleni et al. [80].

Interestingly, the findings of this study emphasized that integrating two unique nanoparticles (which formed a nanocomposite) into the A-2168 silicone enhances the nanocomposite’s hardness more effectively than its counterparts and preserves the silicone’s resiliency against various accelerated aging conditions.

According to Marrega Malavazi et al. [61], the A-2186 silicone demonstrated enhanced hardness compared to other silicone types upon aging. This heightened hardness can be ascribed to its increased filler content and/or the more pronounced molecular weight of the poly(dimethylsiloxane) (PDMS) inherent to A-2186, as opposed to other silicone variations. This reasoning could explain the observed increase in hardness values for the control group in this study across all evaluated conditions relative to their baseline conditions.

The observed elevation in hardness is principally attributed to the post-cure polymerization and crosslinking of the polymer chains following aging conditions. The density of these crosslinks undergoes alterations contingent on the precise aging conditions. As noted by Goiato et al. [62], by the aging period’s end, the material achieves a heightened degree of polymerization. In a related context, Sonnahalli and Chowdhary [79] elucidated that the material’s exposure to specific environmental conditions can lead to changes in the degree of crosslinking, which subsequently and significantly influences its physical and mechanical characteristics.

This research noted an enhanced hardness in silicone incorporated with 2 wt.% TiO_2_ when subjected to accelerated aging conditions. This increase might be attributed to the dispersion of nanoparticles within the silicone elastomer, potentially amplifying the crosslink density and, consequently, the hardness. Another perspective suggests that these nanoparticles could alter the elastic modulus of the silicone elastomer, a view supported by Wang et al. [10] and confirmed by Sonnahalli et al. [22] and Radey et al. [34].

Shakir and Abdul-Ameer [25] proposed that nanofillers fortify the material by serving as multifunctional crosslinks. By establishing resilient hydrogen bonds between their surface hydroxyl groups and the polydimethylsiloxane (PDMS) chains, there is an enhancement in the polymer’s overall crosslink density, rendering it more resilient and rigid. Further observations by Alsmael and Ali [81] highlighted that integrating a titanium nanofiller gradually increases hardness. With escalating nanofiller concentrations, the interactions between the fillers are strengthened, reducing the voids between polymer chains. Such a compact arrangement, especially at increased filler levels, produces a polymer that is notably more resistant to indentation or penetration.

Conversely, Nobrega et al. [32] and Nguyen et al. [57] reported a reduced hardness in silicone after the integration of TiO_2_ and subsequent aging. Their findings differ due to their selection of the MDX4-4210 silicone variant, while the A-2186 type was utilized in this study. Other factors, such as the opacifier quantity and distinct aging conditions, further explain this disparity. In a related context, Sonnahalli and Chowdhary [79] documented a decrease in hardness following the addition of silver nanoparticles. This outcome might be attributed to the inherent characteristics of the nanoparticles and the choice of the M511 silicone type, which differs from the silicone used in this study.

However, upon exposure to various accelerated aging conditions, the silicone category containing 3 wt.% chitosan yielded the highest hardness values in this study. The pronounced hardness might arise from the specific size of the low-molecular-weight chitosan microparticles used. The higher concentration of these microparticles (3 wt.%) in the silicone is also postulated to augment the crosslinking activity within the polymer chains during aging, in accordance with the study of Sonnahalli et al. [22].

Sonnahalli and Chowdhary [79] emphasized that the physical properties of nanoparticles can be significantly influenced by their shape, size, and surface charge. Even though small particle sizes facilitate a better penetration between polymer molecules, a predominant issue with many nanoparticles is their tendency to agglomerate. The dissolution rate of these particles is not only dependent on their chemical and surface characteristics but also on their size. Moreover, the surrounding media they are placed in further influences this rate. If nanoparticles agglomerate to a size that is bigger than the actual size of the polymer particles, it might create voids surrounding these clusters. This can negatively impact the mechanical properties of the material [32].

Han et al. [30] and Zayed et al. [82] pointed out that the inclusion of nanosized oxide particles can indeed augment the structural attributes of the silicone elastomer matrix. However, it is of paramount importance to carefully maintain an optimal concentration of nanofillers. Their elevated surface energy and notable chemical reactivity make this precision necessary: without it, these nanosized oxide particles might tend towards agglomeration. When external forces act upon the silicone elastomer, these agglomerates become areas of heightened stress within the matrix, which in turn jeopardizes the mechanical properties of the silicone elastomer. Furthermore, Han et al. [30] elucidated that, at a particle concentration of 2.0 wt.%, the nano-oxides achieved an exemplary dispersion within the silicone elastomer A-2186. These particles preserved their original dimensions and played a constructive role in elevating the mechanical characteristics of the silicone elastomer A-2186. Conversely, upon increasing the concentration to 3.0 wt.%, a notable fraction of the nano-oxides agglomerated to varying extents, leading to a decline in the mechanical properties of the silicone elastomer A-2186.

Contrary to the findings of this research, Liu et al. [78] suggested that, when microspheres are uniformly distributed within the matrix, the limited gap between the matrix and the microsphere effectively broadens the distance between crosslinking sites. As a result, the deformation of the polymer chains amongst these crosslinking points intensifies, reducing the material’s hardness.

### 4.6. Surface Roughness

Material roughness gauges the subtle inconsistencies in the texture of a surface. The surface roughness average (*R*a) quantifies the variations in a surface’s elevations and depressions, measured in microinches or micrometers. A surface with pronounced deviations is deemed rough, while one with minor variations is seen as smooth. Several methodologies, encompassing optical modalities, surface profilometers, and scanning electron microscopy, are pivotal for precisely evaluating a material’s surface roughness [83].

The surface roughness was assessed, given its recognized correlation with the performance of mechanical components. Imperfections on the surface might act as starting points for cracks or corrosion [51,84].

In this study’s findings, all specimens across the silicone categories showed decreased surface roughness values when exposed to different conditions. Specifically, the 1 wt.% TC specimen showed the most pronounced reduction after UV artificial weathering exposure. However, an exception was noted for the 1 wt.% chitosan–TiO_2_ specimen, which, contrary to the general trend, exhibited an increase in surface roughness in most conditions, as these specimens exhibited a particularly heightened value after being subjected to the sebum condition. The only exception for these specimens was during outdoor weathering, where a decrease in surface roughness was observed.

In this study, the observed smoothness of surfaces of various silicone categories after exposure to different aging conditions aligns with the findings of Mousa et al. [83], who suggested that specimens tend to have smoother and finer surfaces post-weathering. This phenomenon can be attributed to the ongoing polymerization process, which fosters a more organized and comprehensive polymeric chain, resulting in a more polished and delicate surface [62,83,84].

Moreover, nanofillers play a pivotal role in strengthening materials. Specifically, nanoparticles (NPs) act as multifunctional crosslinks, establishing strong hydrogen bonds between surface hydroxyl groups and the polydimethylsiloxane (PDMS) chains. This intricate bonding system enhances the crosslinking density within the polymer, thereby increasing its rigidity and strength. These particles, via crosslinking reactions, amplify the surface energy of the silicone base, creating a matrix structure. In recent research, it has been observed that titanium dioxide nanoparticles contribute positively to the mechanical properties of silicone elastomers. These specific TiO_2_ nanoparticles, utilized as fortifiers for maxillofacial silicones, are distinguished by their minuscule dimensions, vast specific surface area, dynamic capabilities, and efficient interaction with polymers. When introduced into the silicone matrix, they offer enhanced defense against environmental degradation and aging, while concurrently improving the polymer’s inherent physical and mechanical properties. Due to their ability to absorb UV rays, titanium dioxide NPs are frequently employed in nanotechnological applications. Their stability and non-migratory nature within a polymer matrix and sustained thermal stability render them invaluable. To sum up, nanofillers operate as broad-spectrum crosslinking entities, and their addition results in a more polished finish of the material [9,25,34,85].

In this research, integrating nanocomposites into silicone had a synergistic impact on surface roughness after exposure to UV artificial weathering and after six months of outdoor conditions. It is important to reiterate that sunlight exposure negatively affects the structural stability of silicone elastomers. As highlighted earlier, UV radiation causes molecular alterations and intensifies these materials’ inherent crystallinity.

Conversely, the surface roughness showed a noticeable increase when silicone samples with 1 wt.% chitosan–TiO_2_ were immersed in sebum for six months. This change can be attributed to the interaction of sebum’s fatty acids with the silicone. This observation aligns with Eleni et al.’s [80] study, which suggests that certain compounds could be extracted from the sample matrix when exposed to fatty solutions. Furthermore, Hatamleh et al. [37] highlighted that the fatty acids in sebum can affect silicone by breaking chain bonds, leading to the elastomer’s decomposition and making the silicone surface more rugged.

Additionally, Al-Dharrab et al. [51] emphasized that an extended exposure to various storage solutions (for six months) can lead to chemical structure breakdown, resulting in microcracks and surface pits on the material. Such disruptions diminish the binding and surface energies, compromising the thermal resistance of the surface layer and exacerbating other degradation effects. The material’s hydrophobic nature, closely related to its surface appearance (or smoothness), and the storage duration substantially impact its properties.

### 4.7. Limitations and Recommendations

Comparing outcomes across different studies is notably complex due to the significant variations in maxillofacial prosthetic materials, the methodologies adopted in experimental testing, and the diverse set of parameters for simulating aging conditions. Notably, this study employed a distinctive combination of two nanocomposites, a fusion that has not been explored in previous research.

Additionally, in this study, the specimens were subjected to rigorous conditioning, exposing them to environments with acidic sweat, sebum, antibacterial agents, and enhanced lighting conditions, potentially exceeding what might be typically experienced. While one of the fundamental objectives was to isolate and understand the factors affecting the aging process of silicone facial prosthetics, it is noteworthy that these prosthetics, in real-life settings, face a cumulative effect of these factors, each varying in intensity and duration. Nevertheless, this research meticulously analyzed the individual influences of these factors, concluding that, when combined, these conditioning factors exerted the most pronounced degradation on silicone prosthetic materials.

It is paramount to undertake clinical studies to obtain insights into the real-world implications for patients. The inherent mechanical properties of maxillofacial silicone prostheses, which differ depending on their variety and manufacturer, necessitate a targeted and specialized research approach. As it stands, the mechanical attributes of maxillofacial silicone prostheses in a live clinical setting remain an uncharted area of study.

## 5. Conclusions

The following are the conclusions of this research:

1. Exposure to sweat, UV artificial weathering, and natural outdoor weathering significantly impacts the tensile strength across all silicone categories. Despite the significant differences, the silicone samples containing 1 wt.% chitosan–TiO_2_ demonstrated the highest tensile strength after being subjected to various aging conditions.
2. Upon being subjected to accelerated aging conditions, silicone variants containing 1 wt.% TC and 2 wt.% TiO_2_ consistently exhibited superior elongation percentages relative to the other silicone categories that were assessed.
3. All silicone categories demonstrated an increase in tear strength values after being subjected to diverse conditions compared to their baseline conditions. Remarkably, the silicone samples with 1 wt.% chitosan–TiO_2_ showed a significant increase in tear strength, especially after exposure to antibacterial and outdoor conditions.
4. There are highlighted variations in the hardness properties among silicone categories for all conditions, except their baseline conditions. Upon six months of sustained exposure to sebum, the 1 wt.% TC specimen had the lowest hardness value. Concurrently, when exposed to sweat and outdoor weathering conditions, the 3 wt.% chitosan variant presented a pronounced increase in the hardness metrics compared to the 1 wt.% TC and 1 wt.% chitosan–TiO_2_ specimens.
5. All specimens across the silicone categories showed decreased surface roughness values when exposed to different conditions. Specifically, the 1 wt.% TC specimen showed the most pronounced reduction after UV artificial weathering exposure. However, an exception was noted for the 1 wt.% chitosan–TiO_2_ specimen, which exhibited an increase in surface roughness in most conditions, contrary to the general trend.
6. The findings of this study emphasize that integrating two unique nanoparticles (which formed a nanocomposite) into the A-2168 silicone enhances its mechanical properties more effectively than its counterparts and preserves the silicone’s properties against various accelerated aging conditions.

## Figures and Tables

**Table 1:** Monthly average climate data during the outdoor weathering condition in 2022 in Sulaimani City in the Kurdistan Region in northern Iraq.

Date (2022)	Temperature (°C)			Average Humidity (%)	Pressure (m bar)
	Max	Min	Average		
June	37.4	24.6	31.0	28.3	910.1
July	40.7	26.8	33.7	24.2	906.4
August	42.1	28.0	35.0	23.9	908.5
September	36.9	23.3	30.1	29.8	912.3
October	30.5	18.0	24.2	42.8	917.6
November	20.0	9.8	14.9	62.1	919.2
December	14.7	6.6	10.7	65.5	920.2

**Table 2:** Mean values and standard deviations of the tensile strength of silicone categories under different conditions.

Condition	1 wt.% Chitosan–TiO_2_	1 wt.% TC	2 wt.% TiO_2_	3wt.% Chitosan	Control (Zero Nano)	*p*-Value
Baseline (No Weathering)	9.88 ± 0.94	9.53 ± 0.97	9.65 ± 0.92	8.35 ± 0.57	9.35 ± 0.60	**0.07**
Sweat (6 Months)	10.23 ± 0.70 ^a^	8.76 ± 0.92	9.88 ± 0.74	9.18 ± 0.57	9.88 ± 0.97	**0.05**
Sebum (6 Months)	10.53 ± 1.81	10.76 ± 0.68 *	10.53 ± 1.05	9.82 ± 0.90	10.53 ± 1.46	**0.80**
UV Weathering (720 h)	11.29 ± 1.01 ^abcd^	8.94 ± 0.53	9.71 ± 1.16	9.00 ± 0.26	8.88 ± 0.70	**0.00**
Outdoor Weathering (6 Months)	11.29 ± 0.85 ^abcd^	8.71 ± 0.79	8.94 ± 0.64	8.76 ± 0.92	8.53 ± 1.98	**0.005**
Antibacterial (30 h)	10.35 ± 1.13	9.47 ± 1.20	9.71 ± 1.06	8.82 ± 0.91	9.76 ± 1.52	**0.386**
*p*-Value	**0.29**	**0.01**	**0.24**	**0.08**	**0.21**	

^a^ Compared to 1 wt.% TC. ^b^ Compared to 2 wt.% TiO_2_. ^c^ Compared to 3 wt.% chitosan. ^d^ Compared to the control (zero nano). Different superscript letters in the same row indicate the only significant differences presented within properties (*p* < 0.05) after applying a one-way ANOVA and (Dunnett’s T3 and Tukey’s HSD) multiple comparison tests between specimens. The symbol (*) in the same column indicates significant differences between the groups (*p* < 0.05) when compared to their baseline conditions after applying independent *t*-tests.

**Table 3:** Mean values and standard deviations of the elongation percentages of silicone categories with different conditions.

Condition	1 wt.% Chitosan–TiO_2_	1 wt.% TC	2 wt.% TiO_2_	3 wt.% Chitosan	Control (Zero Nano)	*p*-Value
Baseline (No Weathering)	422.63 ± 55.88 ^ab^	587.6 ± 39.49 ^c^	602.35 ± 72.90 ^c^	415.56 ± 37.40	628.65 ± 110.26	**0.00**
Sweat (6 Months)	373.21 ± 35.97 ^abd^	575.63 ± 31.16 ^cd^	526.09 ± 64.11 ^c^	377.32 ± 42.47 ^d^	461.14 ± 43.59 *	**0.00**
Sebum (6 Months)	389.03 ± 79.51 ^ab^	519.38 ± 27.36 ^c^	541.24 ± 34.70 ^c^	379.12 ± 41.26 ^d^	485.23 ± 65.54 *	**0.00**
UV Weathering (720 h)	418.32 ± 42.85 ^a^	582.05 ± 60.14	489.51 ± 60.40	445.79 ± 84.47	516.44 ± 49.11	**0.004**
Outdoor Weathering (6 Months)	410.14 ± 38.10 ^a^	577.95 ± 55.13 ^d^	504.90 ± 86.85	448.00 ± 87.70	426.92 ± 108.11 *	**0.02**
Antibacterial (30 h)	409.87 ± 44.42 ^abd^	551.15 ± 72.00 ^c^	574.82 ± 90.06 ^c^	410.61 ± 84.47 ^d^	573.75 ± 112.04	**0.002**
*p*-Value	**0.65**	**0.29**	**0.15**	**0.27**	**0.01**	

^a^ Compared to 1 wt.% TC. ^b^ Compared to 2 wt.% TiO_2_. ^c^ Compared to 3 wt.% chitosan. ^d^ Compared to the control (zero nano). Different superscript letters in the same row indicate the only significant differences presented within properties (*p* < 0.05) after applying a one-way ANOVA and (Dunnett’s T3 and Tukey’s HSD) multiple comparison tests between specimens. The symbol (*) in the same column indicates significant differences between groups (*p* < 0.05) when compared to their baseline conditions after applying independent *t*-tests.

**Table 4:** Mean values and standard deviations of the tear strength test of the silicone types under different conditions.

Condition	1 wt.% Chitosan–TiO_2_	1 wt.% TC	2 wt.% TiO_2_	3 wt.% Chitosan	Control (Zero Nano)	*p*-Value
Baseline (No Weathering)	29.33 ± 3.90	27.33 ± 4.62	33.33 ± 1.57 ^c^	26.22 ± 1.69	30.44 ± 2.30	**0.01**
Sweat (6 Months)	29.56 ± 4.49	31.78 ± 4.35	34.22 ± 5.18 ^c^	26.67 ± 2.08 ^d^	34.22 ± 4.54	**0.05**
Sebum (6 Months)	33.33 ± 7.20	33.56 ± 5.35	30.44 ± 4.94	33.33 ± 3.42 *	33.78 ± 7.44	**0.89**
UV Weathering (720 h)	26.44 ± 3.96	32.00 ± 6.87	29.33 ± 3.39	26.00 ± 2.56	29.11 ± 4.61	**0.253**
Outdoor Weathering (6 Months)	34.44 ± 2.36 *^c^	35.56 ± 7.49	33.33 ± 3.85	27.11 ± 2.56	34.22 ± 4.80	**0.064**
Antibacterial (30 h)	36.44 ± 4.47 *	31.11 ± 5.72	30.00 ± 5.39	28.22 ± 2.79	28.89 ± 8.28	**0.191**
*p*-Value	**0.02**	**0.39**	**0.34**	**0.00**	**0.43**	

^a^ Compared to 1 wt.% TC. ^b^ Compared to 2 wt.% TiO_2_. ^c^ Compared to 3 wt.% chitosan. ^d^ Compared to the control (zero nano). Different superscript letters in the same row indicate the only significant differences presented within properties (*p* < 0.05) after applying a one-way ANOVA and (Dunnett’s T3 and Tukey’s HSD) multiple comparison tests between specimens. The symbol (*) in the same column indicates significant differences between groups (*p* < 0.05) when compared to their baseline conditions after applying independent *t*-tests.

**Table 5:** Mean values and standard deviations of the hardness test of the silicone categories under different conditions.

Condition	1 wt.% Chitosan–TiO_2_	1 wt.% TC	2 wt.% TiO_2_	3 wt.% Chitosan	Control (Zero Nano)	*p*-Value
Baseline (No Weathering)	36.62 ± 2.53	36.19 ± 0.90	35.75 ± 0.97	37.05 ± 2.30	34.75 ± 1.49	**0.325**
Sweat (6 Months)	40.42 ± 2.62 ^a^	36.42 ± 1.68 ^c^	37.57 ± 1.52	40.13 ± 1.81 *	37.1 ± 1.34 *	**0.006**
Sebum (6 Months)	40.43 ± 1.79 ^a^	35.96 ± 1.18 ^bcd^	38.41 ± 0.52 *	39.25 ± 0.79	39.556 ± 0.89 *	**0.000**
UV Weathering (720 h)	38.50 ± 2.70	37.65 ± 1.96	38.88 ± 1.21 *	40.84 ± 1.62 *^d^	36.82 ± 0.65 *	**0.021**
Outdoor Weathering (6 Months)	37.36 ± 2.64 ^c^	37.58 ± 0.50	38.6 ± 0.59 *	40.76 ± 2.61 *	38.23 ± 1.12 *	**0.046**
Antibacterial (30 h)	39.43 ± 1.91 ^ad^	36.53 ± 1.12 ^c^	38.4 ± 0.80 *	39.4 ± 1.04 ^d^	36.73 ± 0.95 *	**0.002**
*p*-Value	**0.088**	**0.227**	**0.000**	**0.03**	**0.000**	

^a^ Compared to 1 wt.% CT. ^b^ Compared to 2 wt.% TiO_2_. ^c^ Compared to 3 wt.% chitosan. ^d^ Compared to the control (zero nano). Different superscript letters in the same row indicate the only significant differences presented within properties (*p* < 0.05) after applying a one-way ANOVA and (Dunnett’s T3 and Tukey’s HSD) multiple comparison tests between specimens. The symbol (*) in the same column indicates significant differences between groups (*p* < 0.05) when compared to their baseline conditions after applying independent *t*-tests.

**Table 6:** Mean values and standard deviations of the surface roughness of the silicone categories under different conditions.

Condition	1 wt.% Chitosan–TiO_2_	1 wt.% TC	2 wt.% TiO_2_	3 wt.% Chitosan	Control (Zero Nano)	*p*-Value
Baseline (No Weathering)	0.27 ± 0.001 ^abcd^	0.27 ± 0.0008 ^cd^	0.27 ± 0.0008 ^cd^	0.68 ± 0.001 ^d^	0.64 ± 0.0008	**0.00**
Sweat (6 Months)	0.34 ± 0.0008 *^abcd^	0.21 ± 0.0008 *^bcd^	0.28 ± 0.0008 *^cd^	0.64 ± 0.0005 *^d^	0.27 ± 0.0008 *	**0.00**
Sebum (6 Months)	0.80 ± 0.0008 *^abcd^	0.24 ± 0.0008 *^bcd^	0.22 ± 0.0004 *^cd^	0.60 ± 0.0008 *^d^	0.59 ± 0.0005 *	**0.00**
UV Weathering (720 h)	0.48 ± 0.0005 *^abcd^	0.20 ± 0.0007 *^bcd^	0.26 ± 0.0005 *^cd^	0.42 ± 0.0008 *^d^	0.60 ± 0.0008 *	**0.00**
Outdoor Weathering (6 Months)	0.24 ± 0.0008 *^abcd^	0.29 ± 0.005 *^bcd^	0.22 ± 0.0005 *^cd^	0.61 ± 0.0008 *^d^	0.68 ± 0.0005 *	**0.00**
Antibacterial (30 h)	0.47 ± 0.0005 *^abcd^	0.24 ± 0.0008 *^bcd^	0.28 ± 0.0005 *^cd^	0.49 ± 0.0008 *^d^	0.44 ± 0.0008 *	**0.00**
*p*-Value	**0.000**	**0.000**	**0.000**	**0.000**	**0.000**	

^a^ Compared to 1 wt.% TC. ^b^ Compared to 2 wt.% TiO_2_. ^c^ Compared to 3 wt.% chitosan. ^d^ Compared to the control (zero nano). Different superscript letters in the same row indicate the only significant differences presented within properties (*p* < 0.05) after applying a one-way ANOVA and (Dunnett’s T3 and Tukey’s HSD) multiple comparison tests between specimens. The symbol (*) in the same column indicates significant differences between groups (*p* < 0.05) when compared to their baseline conditions after applying independent *t*-tests.

## Data Availability

The data supporting this study’s findings are available upon request from the corresponding author (Al-Kadi, F.K.).
